# Nanodiamond-DGEA peptide conjugates for enhanced delivery of doxorubicin to prostate cancer

**DOI:** 10.3762/bjnano.5.107

**Published:** 2014-07-01

**Authors:** Amanee D Salaam, Patrick Hwang, Roberus McIntosh, Hadiyah N Green, Ho-Wook Jun, Derrick Dean

**Affiliations:** 1 Department of Biomedical Engineering, University of Alabama at Birmingham (UAB), 1530 3rd Avenue South, Birmingham, AL 35294, USA; 2 Department of Materials Science and Engineering, University of Alabama at Birmingham (UAB), 1530 3rd Avenue South, Birmingham, AL 35294, USA; 3 Department of Materials Science and Engineering, Tuskegee University (TU), 1200 W Montgomery Rd, Tuskegee, AL 36088, USA

**Keywords:** DGEA peptide, doxorubicin, nanodiamond, prostate cancer, targeted drug delivery

## Abstract

The field of nanomedicine has emerged as an approach to enhance the specificity and efficacy of cancer treatments as stand-alone therapies and in combination with standard chemotherapeutic treatment regimens. The current standard of care for metastatic cancer, doxorubicin (DOX), is presented with challenges, namely toxicity due to a lack of specificity and targeted delivery. Nano-enabled targeted drug delivery systems can provide an avenue to overcome these issues. Nanodiamonds (ND), in particular, have been researched over the past five years for use in various drug delivery systems but minimal work has been done that incorporates targeting capability. In this study, a novel targeted drug delivery system for bone metastatic prostate cancer was developed, characterized, and evaluated in vitro. NDs were conjugated with the Asp–Gly–Glu–Ala (DGEA) peptide to target α_2_β_1_ integrins over-expressed in prostate cancers during metastasis. To facilitate drug delivery, DOX was adsorbed to the surface of the ND-DGEA conjugates. Successful preparation of the ND-DGEA conjugates and the ND-DGEA+DOX system was confirmed with transmission electron microscopy, hydrodynamic size, and zeta potential measurements. Since traditional DOX treatment regimens lack specificity and increased toxicity to normal tissues, the ND-DGEA conjugates were designed to distinguish between cells that overexpress α_2_β_1_ integrin, bone metastatic prostate cancers cells (PC3), and cells that do not, human mesenchymal stem cells (hMSC). Utilizing the ND-DGEA+DOX system, the efficacy of 1 µg/mL and 2 µg/mL DOX doses increased from 2.5% to 12% cell death and 11% to 34% cell death, respectively. These studies confirmed that the delivery and efficacy of DOX were enhanced by ND-DGEA conjugates. Thus, the targeted ND-DGEA+DOX system provides a novel approach for decreasing toxicity and drug doses.

## Introduction

Prostate cancer is the most frequently diagnosed malignancy in men [1]. Typically the disease is slow growing, but in some cases it progresses to an aggressively metastatic state. When prostate cancer becomes metastatic, the current standard of care is chemotherapy, which involves the use of toxic anticancer drugs, like doxorubicin (DOX), to treat cancers by inducing apoptosis. DOX has had high success rates with treating prostate cancer [2]. However, it can cause major side effects such as hair loss, nausea [2–3], and cardiomyopathy (weakening of the heart muscle) [4–5]. Like most clinical chemotherapy regimens, DOX lacks specificity (or targeting) and eradicates most rapidly dividing cells (e.g., hair, immune, and many other types of normal cells). As a result, there is a need to improve treatment specificity, efficacy, and toxicity by incorporating mechanisms for targeted delivery of chemotherapeutics.

Nanomedicine has become a viable solution for the specificity and toxicity problems with current chemotherapy treatment regimens [6–9]. Nanoparticles have facilitated tumor targeting and drug delivery in a variety of tumor types [6–9]. Currently, there are several clinically approved nanoparticle-based cancer drugs using liposomes, nanoparticle albumin-bound (nab) technology, dendrimers, polymeric, carbon, and metal nanoparticles [6,8]. Gold nanorods, iron magnetic nanoparticles, polymer nanospheres, lipids, and gadolinium oxide nanoparticles are also being utilized to strategically target prostate and various other cancers [10–16]. These techniques have proven the importance of targeting for improved chemotherapeutic efficacy, but there can be limitations with biocompatibility, delivery due to size, and bioavailability.

In contrast to the aforementioned nanoparticle systems, nanodiamond particles (ND) possess advantageous properties such as rich surface chemistry for conjugating targeting molecules, high surface area for loading drugs, and the ability to act as transmembrane carriers [17–20]. NDs have been used as a vehicle for targeted drug delivery platforms [16,21–25]. NDs have already been shown to improve the efficacy of DOX for treating breast cancers and gliomas [26–28]. Even though pre-clinical work has been done with targeted NDs, the efficacy enhancement properties of NDs for targeted metastatic prostate cancer treatments has not been previously reported.

Prostate cancers have been known to exhibit various aberrations, such as integrin α and β subunits, depending on the stage of progression. Integrin α and β subunits α_6_, β_1_, β_3_ and β_6_ are up-regulated in metastatic cancers [29], while α_2_ is down-regulated initially then up-regulated as disease progresses [29–30]. High expression of the α_2_β_1_ integrin has been correlated with tumor progression in a number of cancers [31–33]. The α_2_β_1_ integrin is a receptor mainly for type I collagens, laminins, E-cadherin, and matrix metalloproteinase 1 [31]. α_2_β_1_ integrins have been proven to be up-regulated in bone metastatic prostate cancer cells [32–33]. Particularly, PC3 human bone metastatic prostate cancer cell lines have the highest expression of α_2_β_1_ integrins when compared to other metastatic cell lines CWR-22 and LNCaP [31]. The over-expression of α_2_β_1_ integrins in PC3 can be harnessed as a target for a drug delivery platform.

The toxicity of DOX can be decreased by increasing the interaction between the drug and cancer cells. Since α_2_β_1_ integrins are over-expressed in bone metastatic prostate cancers, targeted drug delivery with a ligand that interacts with these integrins should allow for increased accumulation of drug systems in cancer cells versus normal cells or tissues. Asp–Gly–Glu–Ala (DGEA) peptide has been identified as a binding peptide for the α_2_β_1_ integrins; it corresponds to residues 435 to 438 of the type I collagen [34]. To our knowledge, current literature does not report the use of DGEA for improving drug delivery in cancers over-expressing α_2_β_1_, despite the abilities of DGEA to facilitate in vivo imaging of α_2_β_1_ integrins in cancers [31,35]. Thus in the current work, we developed a novel ND meditated drug delivery system to increase specificity of DOX by utilizing DGEA to target the α_2_β_1_ integrins overexpressed in metastatic prostate cancers. ND-DGEA conjugates and the ND-DGEA+DOX system were synthesized and evaluated for multifunctional applications (i.e, targeting and drug delivery). We show significantly improved efficacy and toxicity of DOX by using ND-DGEA conjugates to deliver DOX therapy to prostate cancer cells.

## Experimental

### Materials

All materials, buffers, and reagents were purchased and used as received. ND hard gel (≈20% water) was purchased from NanoCarbon Research Institute (Osaka, Japan). DGEA peptide with a fluorescein isothiocyanate (FITC) tag attached via a lysine residue was purchased from Celtek Peptides (Nashville, TN). 1-Ethyl-3-(3-dimethylaminopropyl) carbodiimide (EDAC) and *N*-hydroxysulfosuccinimide (sulfo-NHS) were purchased from Sigma Aldrich (St. Louis, MO). Activation (pH 5.5), coupling (pH 8.5), and washing/storage (pH 7.4) buffers were purchased from Ocean NanoTech (Springdale, AR). DOX was purchased from LC Laboratories (Woburn, MA).

### Synthesis of ND-DGEA conjugates

**Scheme 1:**
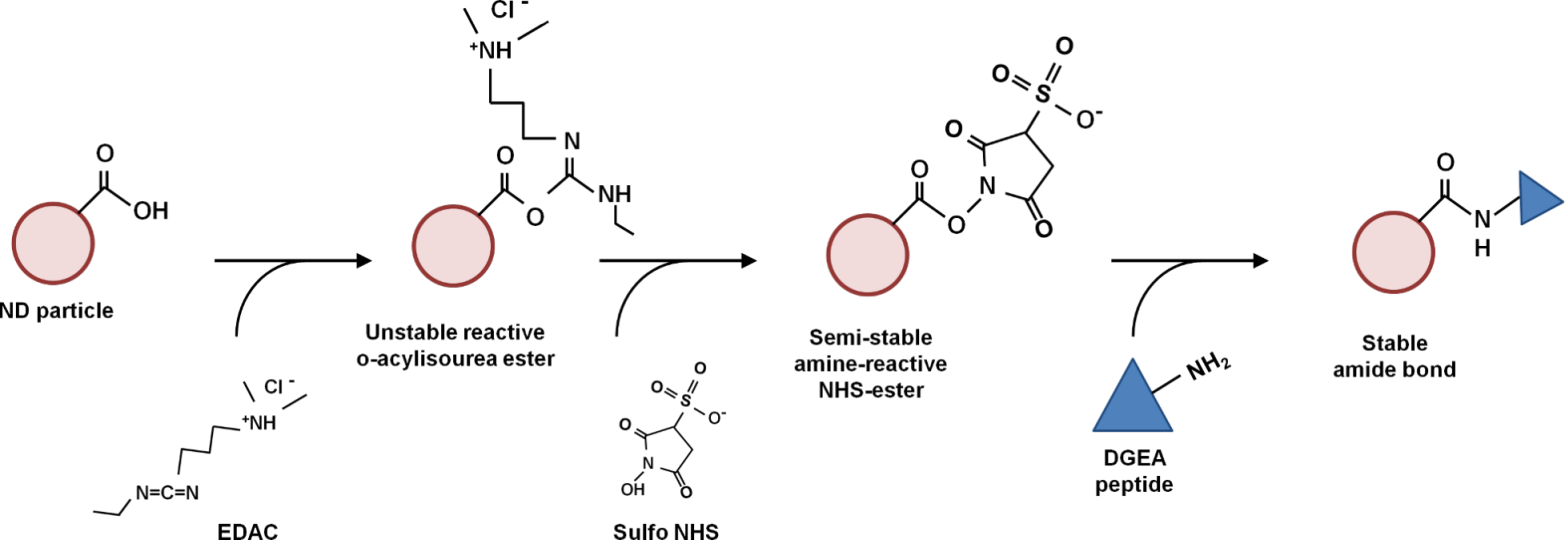
Nanodiamond-DGEA peptide conjugation. The carbodiimide reaction used EDAC and sulfo NHS to form a stable amide bond between the free amine group on DGEA peptide and the carboxyl group on the surfaces of NDs covalently linking the two materials.

NDs were conjugated with DGEA using carbodiimide chemistry as shown in Scheme 1. With this technique, EDAC was used to activate carboxylic groups on the ND surface. Sulfo-NHS was used to form a stable amide bond between the –COOH groups on the NDs and the free NH_2_ groups on DGEA. Briefly, 200 µL of ND colloid (5 mg/mL in distilled water) was added with 100 µL of activation buffer and continuously mixed for 5 min at ambient temperature. Then, 50 µL of EDAC (1 mg/mL in activation buffer) and 50 µL of sulfo-NHS (1 mg/mL in activation buffer) were added and continuously mixed for 30 min at ambient temperature. Next, 200 µL of coupling buffer and 50 µL of DGEA peptide solution (2 mg/mL) were added, and the solution was continuously mixed for 2 h. The solution was then centrifuged at 10,000 rpm for 10 min. The DGEA peptide functionalized ND was washed three times with 200 µL of washing/storage buffer, centrifuging after each wash. The ND-DGEA conjugates were then lyophilized for characterization and stored at 4 °C.

### Analysis of peptide conjugation efficiency

The peptide conjugation efficiency was determined using a microplate reader (Biotek Synergy 2, Winooski, VT). In this technique, absorbance spectra for various concentrations of DGEA were obtained from 350 to 650 nm. A linear relationship between the peak absorbance (490 nm) of DGEA and concentration was observed in accordance with Beer–Lambert’s Law. The mass of un-conjugated peptide in the supernatant and wash buffer were quantified using linear regression. The percentage of peptide conjugation efficiency was determined using the following Equation 1, where *M*_P_ was the mass of peptide added and *M*_UP_ was the mass of un-conjugated peptide:

[1]
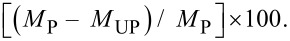


The amount of conjugated peptide was equivalent to difference of *M*_P_ and *M*_UP_. The percentage of conjugation capacity of the NDs was also calculated using the following Equation 2, where *M*_ND_ was the mass of NDs added:

[2]
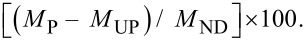


### Synthesis of ND-DGEA+DOX system

**Scheme 2:**
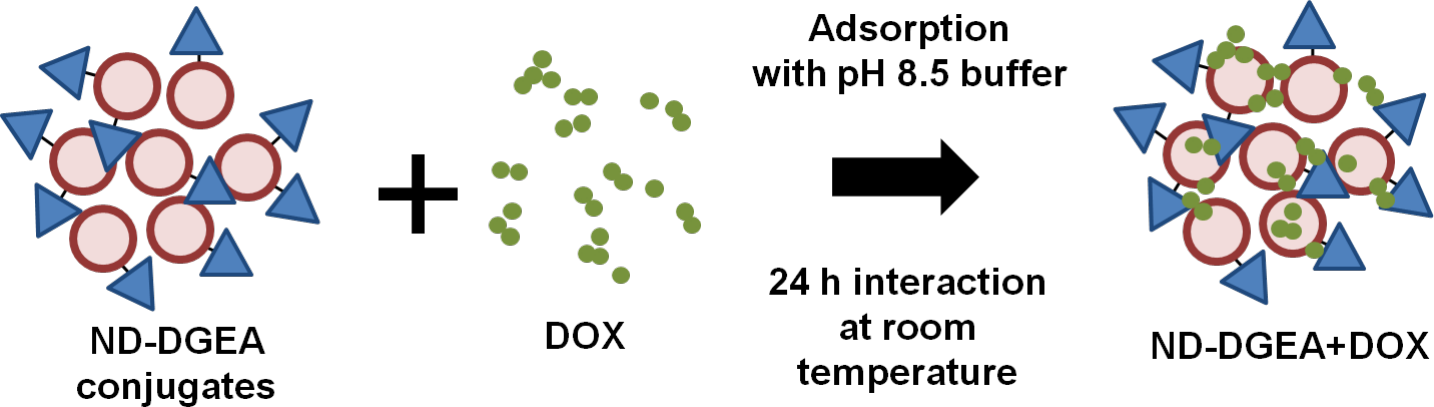
Preparation of ND-DGEA+DOX system. After conjugation of DGEA peptide to NDs, DOX was adsorbed to the ND-DGEA conjugates using a pH 8.5 buffer and interacting for 24 h at room temperature.

After conjugating ND with DGEA, DOX was adsorbed to the ND-DGEA conjugates in alkaline conditions (pH 8.5) based on prior optimization of DOX modified NDs (Scheme 2). The ND-DGEA conjugates were resuspended in 400 µL distilled water using an ultrasonic water bath. Then, 400 µL activation buffer were added to the ND-DGEA suspension and the mixture was continuously mixed for 30 min. 500 µL of DOX (1 mg/mL) was added, and the mixture was continuously mixed for an additional hour. Last, 400 µL of the pH 8.5 coupling buffer was added and the DOX and ND-DGEA were interacted for 24 h at room temperature. The quantity of adsorbed DOX was determined using absorbance spectra for various concentrations of DOX from 350 to 650 nm and linear regression techniques.

### Characterization of ND-DGEA conjugates and ND-DGEA+DOX system

The ND-DGEA conjugates and ND-DGEA+DOX system were characterized to confirm successful bonding of ND to DGEA and adsorption of DOX, respectively. Transmission electron microscopy (TEM, FEI Tecnai T12) was used to qualitatively confirm the conjugation and adsorption. Fourier transform infrared spectroscopy (FTIR, Nicolet Thermo Scientific) was used to confirm the chemical bonding of ND to DGEA and the presence of DOX on the ND-DGEA surface; spectra were collected from 400–3500 cm^−1^ at ambient temperature in attenuated total reflectance mode (ATR) with 64 scans per sample. A Zeta-sizer Nano ZS (Malvern) was used to measure the zeta potential and hydrodynamic size of the ND before and after modification with DGEA and DOX; samples were prepared at a concentration of 200 µg/mL.

### Cell culture

Human bone metastatic prostate cancer cells (PC3) and mesenchymal stem cells (hMSC) were acquired from American Type Culture Collection (ATCC, Manassas, VA). PC3 and hMSC cells were grown in RPMI 1640 (Thermo Scientific, Waltham, MA) and Dulbecco's Modified Eagle's Medium (DMEM, Corning, Manassas, VA) media, respectively. The media was supplemented with 10% fetal bovine serum (FBS, Atlanta Biologicals Inc., Atlanta, GA) and 1% penicillin/streptomycin/anphotericin (Fisher Scientific, Hampton, NH) at 37 °C and 5% CO_2_ in a humidiﬁed incubator. The cell lines were cultured in T75 flasks until confluent before use in experiments. All hMSCs and PC3 cells were seeded in 48 well plates and allowed to attach/proliferate for 24 h prior to exposure to 32h treatment regimens.

### Evaluation of ND-DGEA targeting

The effects of DGEA peptide on cell targeting were investigated to determine the optimal parameters for treatment regimens. The cells were exposed to 10 µg/mL ND-DGEA for 32 h. Then, cells were washed three times with phosphate buffered saline (PBS) to remove residual ND-DGEA that had not been attached or internalized. DGEA peptide was synthesized with a fluorescent label (fluorescein isothiocyanate, FITC) to allow for visualization. FITC has an excitation and emission wavelengths of approximately 495 nm and 519 nm, respectively. The interaction between the cells and ND-DGEA was observed with fluorescent microscopy using a blue filter, which covers an excitation wavelength range between 420 and 495 nm.

### In vitro evaluation of ND-DGEA+DOX system

The ND-DGEA+DOX system was evaluated for efficacy with PC3 cells. Cells were exposed to no treatment (control), free DOX, ND+DOX, ND-DGEA+DOX, and DGEA+DOX for 32 h in serum free media since serum-supplemented media contains proteins that hinder drug delivery and provides nutrients that cause to cells to become less sensitive to treatment. To ensure that the effects of the drug delivery system were due to the combination of enhanced targeting and drug delivery instead of potential toxicity of ND or DGEA peptide, PC3 cells were also exposed to ND, ND-DGEA, and free DGEA. The cell viability was quantified with a 3-(4,5-dimethylthiazol-2-yl)-5-(3-carboxymethoxyphenyl)-2-(4-sulfophenyl)-2*H*-tetrazolium (MTS, Promega, Madison, WI) assay. Briefly, MTS assay reagent was added to the cells, and the plates were incubated for 2 h. The absorbance at 490 nm was read with a microplate reader. Cell viability was represented as percentages in reference to the control.

### Statistical analysis

Experiments were performed in triplicate. Data were represented as average with standard deviation. The means were compared with a student’s *t*-test, and *p*-values of 0.05 or less were statistically significant.

## Results and Discussion

### Characterization of ND-DGEA conjugates and ND-DGEA+DOX system

**Figure 1:**
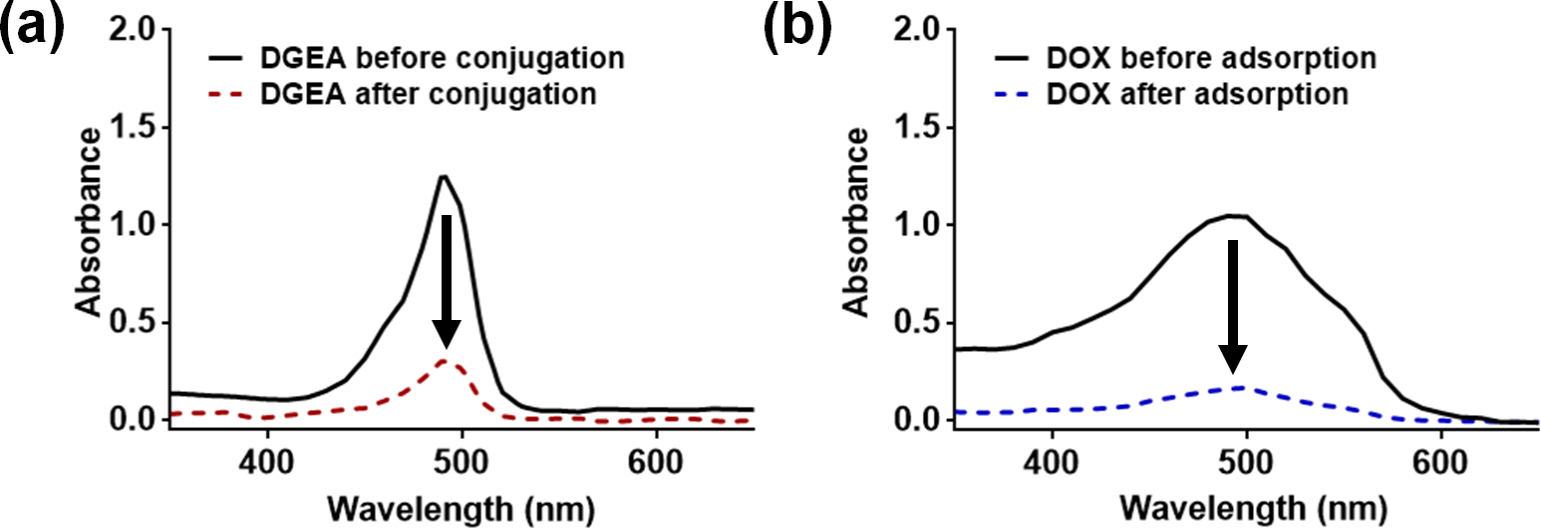
Absorbance spectra for (a) DGEA before and after conjugation with ND and (b) DOX before and after adsorption to ND-DGEA conjugates. 100 µg of DGEA was added prior to conjugation, and about 25 µg remained in the supernatant. Approximately 100 µg of the added 500 µg DOX remained in the supernatant after facilitating adsorption to ND-DGEA.

ND-DGEA conjugates were successfully synthesized utilizing a carbodiimide reaction between the carboxyl groups on the surface of the NDs and the free amine groups on the peptide. The absorbance spectra shown in Figure 1a were taken of DGEA peptide before and after conjugation to NDs. The absorbance peak decreased after modification, indicating that the peptide had been conjugated to the NDs. Based on peptide concentration curves and linear regression, it was determined that about 25 µg of the added 100 µg of DGEA peptide was un-conjugated and remained in the supernatant. The addition of 1 mg ND with the DGEA peptide dictated that the NDs had the capacity to covalently attach 3.8 wt % of peptide. In addition, a peptide conjugation efficiency of 75% was achieved.

**Figure 2:**
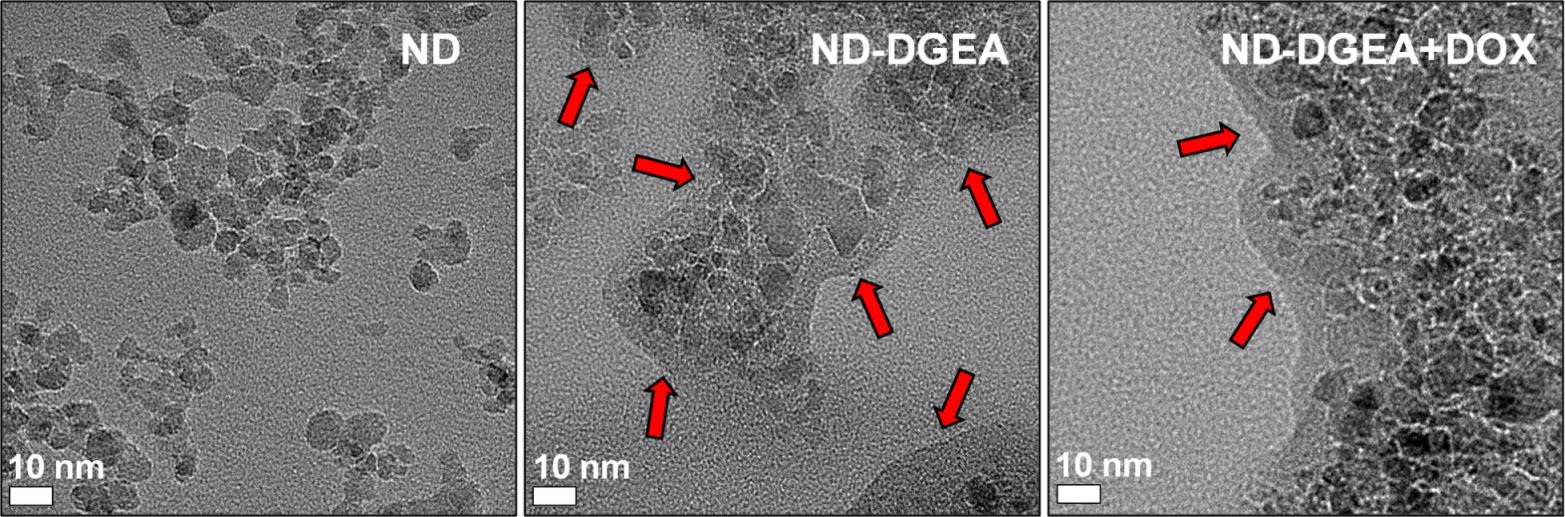
Transmission electron microscope images of ND, ND-DGEA, and ND-DGEA+DOX. Arrows identify the layer of DGEA or DGEA and DOX surrounding the NDs.

**Figure 3:**
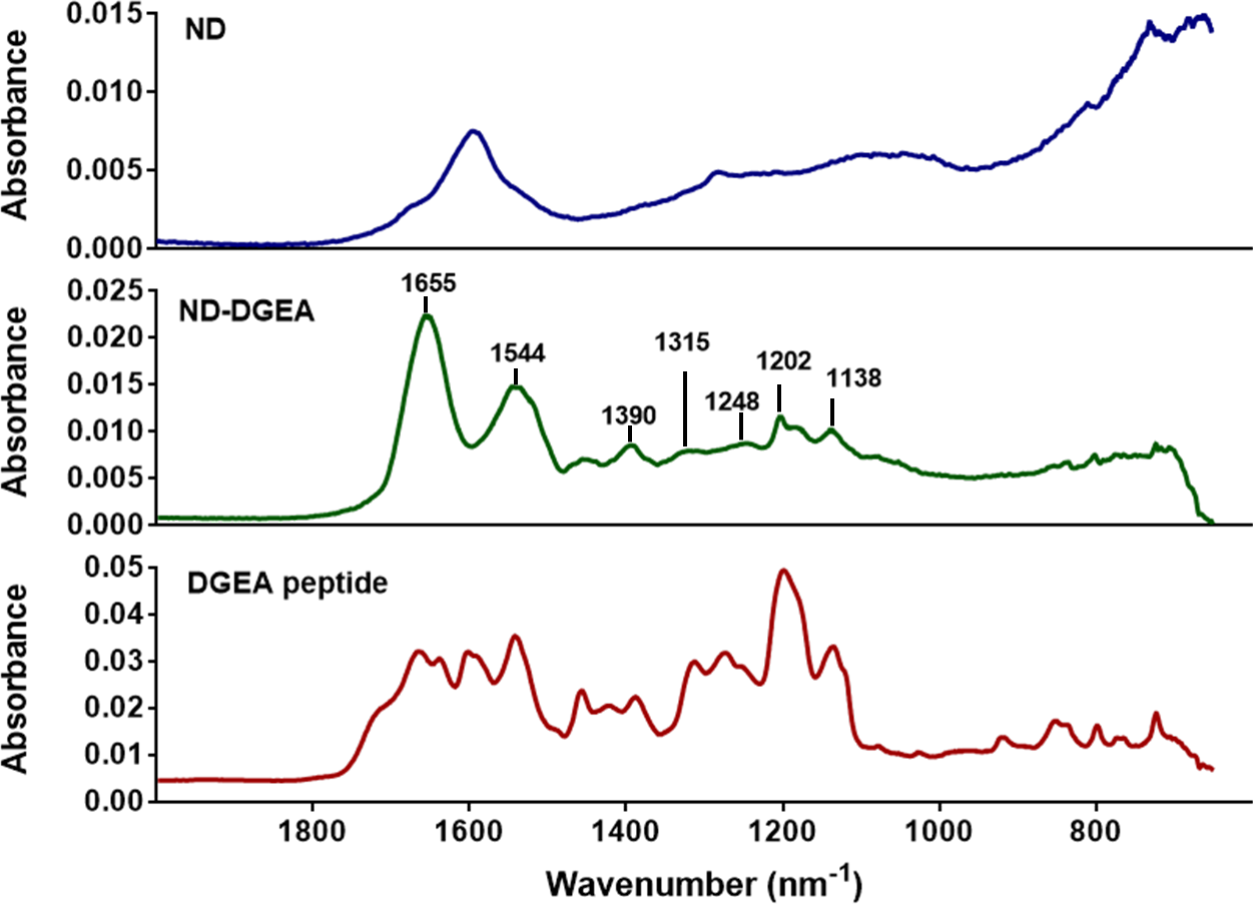
FTIR spectra of ND, ND-DGEA conjugates, and DGEA peptide. The presence of characteristic peaks for DGEA peptide in the spectrum for the ND-DGEA conjugates confirmed successful conjugation.

TEM qualitatively confirmed the conjugation. In Figure 2, the TEM image for ND-DGEA displayed a 10 nm layer of conjugated DGEA surrounding the NDs, as ND and DGEA were the only solid materials were added during synthesis. In addition, several washes were performed after synthesis prior to imaging to remove any residual un-conjugated peptide. In comparison to the pure NDs, there was increased aggregation in the DGEA modified particles, which was mainly due to the covalent bonding of the peptide to the surface. FTIR confirmed that the layer was DGEA as several characteristic peaks for DGEA were represented in the ND-DGEA spectrum (Figure 3). Particularly, there were peaks due to C–N stretching in primary or secondary amines of amino acids between 1130 and 1390 cm^–1^, carbonyl stretching in the amide I bonds (1655 cm^–1^), and NH bending of the primary amine (1544 cm^–1^). There was also a broadening of the 1544 cm^–1^ peak (amide II), signifying successful conjugation as additional amide bonds were formed between the carboxyl groups on the NDs and the free amines on the peptide.

Next, the ND-DGEA+DOX system was prepared by physically adsorbing DOX to the already synthesized ND-DGEA conjugates. After successful adsorption, the peak absorbance in the DOX spectra in Figure 1b decreased to a value of approximately 100 µg by linear regression, suggesting that the ND-DGEA conjugates adsorbed approximately 400 µg of the added 500 µg DOX; this correlated to a 20% loading of DOX on the NDs and 80% DOX loading efficiency. Successful preparation of the ND-DGEA+DOX system was also visually confirmed with TEM (Figure 2). A 15 nm layer was observed surrounding the DGEA and DOX modified NDs, in contrast to the 10 nm layer observed for the NDs modified with only DGEA peptide.

**Figure 4:**
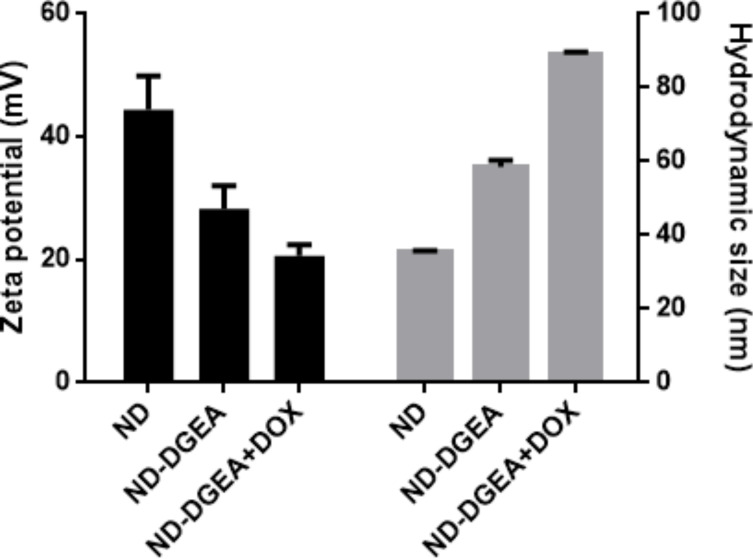
Zeta potential and hydrodynamic size of ND, ND-DGEA, and ND-DGEA+DOX.

After synthesizing the ND-DGEA conjugates and the ND-DGEA+DOX system, hydrodynamic size and zeta potential measurements were performed on both materials. Hydrodynamic size and zeta potential are particularly important for drug delivery applications as they give representations of size limitations and potential colloidal stability issues of a system. NDs permeate the cell membrane by endocytosis [20,36–37]. Through this pathway, NDs can effectively deliver drugs but aggregate particle size should be less than 100 nm and zeta potential values greater than 30 mV in magnitude for good stability of particles in solution. The hydrodynamic size increased from 35 nm for pure NDs to 59 nm for the ND+DGEA conjugates to 89 nm for the ND-DGEA+DOX system (Figure 4). This trend correlated well with what was observed with TEM where the width of the layer surrounding the NDs increased from 10 nm for the ND-DGEA conjugates to approximately 15 nm for the ND-DGEA+DOX system (Figure 2). The increased aggregation observed with TEM was also confirmed with the hydrodynamic measurements, but the size of the ND-DGEA+DOX system was within the optimal range for drug delivery (<100 nm).

The zeta potential indicated that the colloidal stability decreased with each modification of the NDs. At a prepared concentration of 200 µg/mL, the zeta potentials for pure NDs, ND-DGEA, and ND-DGEA+DOX were 44 mV, 28 mV, and 21 mV, respectively (Figure 4). The change in zeta potential was an indicator of successful modification as several researchers have confirmed a decrease in zeta potential after nanoparticle modification [11,26]. Although the zeta potentials for the ND-DGEA conjugates and ND-DGEA+DOX system decreased below 30 mV, both the conjugates and systems were stable at dilute concentrations in aqueous solutions for several days.

### ND-DGEA selective targeting

**Figure 5:**
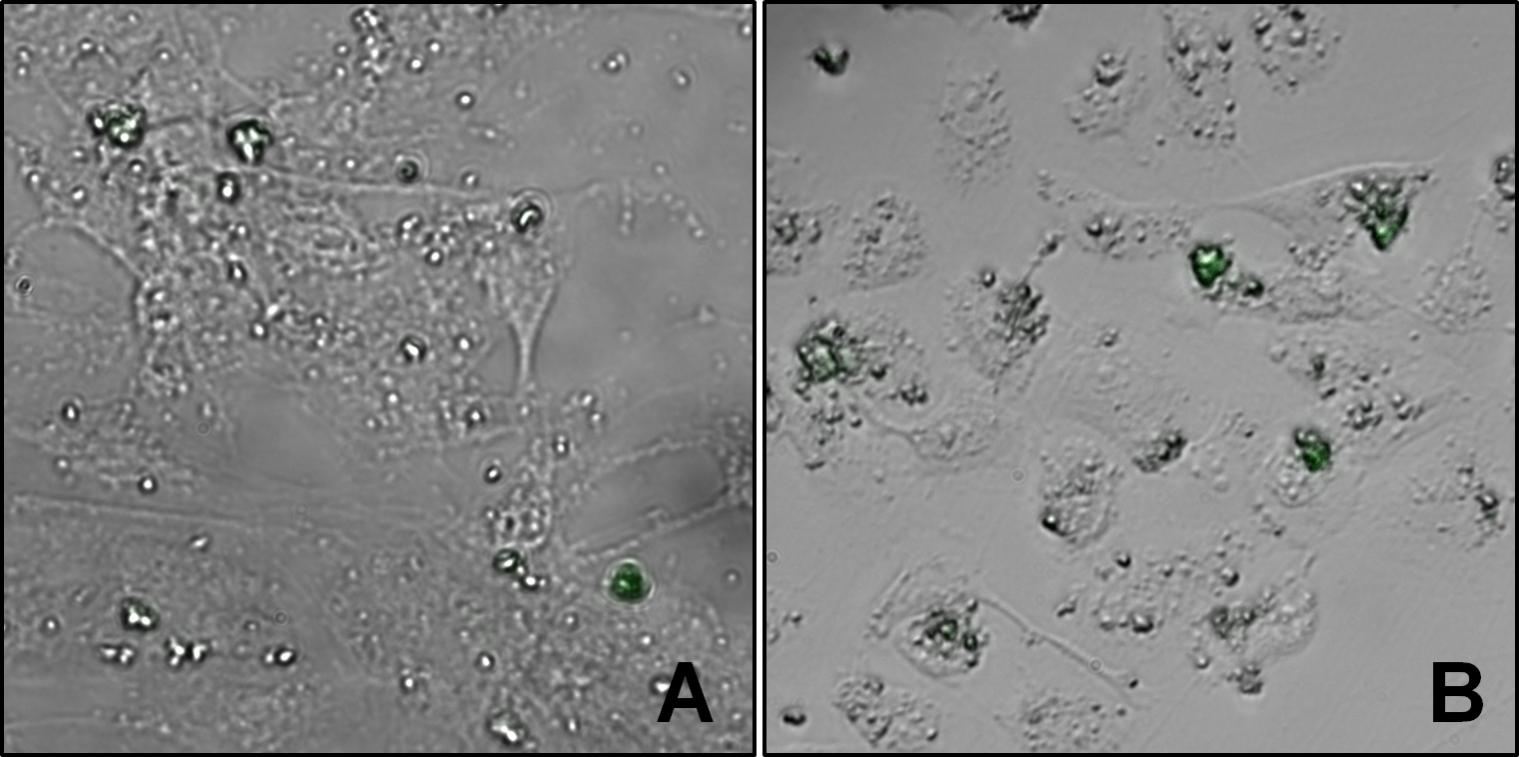
Representative merged bright field and fluorescent microscopy images of hMSCs (A) and PC3 cells (B) after 32 h exposure to 10 µg/mL ND-DGEA conjugates. After treatment, cells were washed three times with PBS to remove unattached or internalized ND-DGEA and imaged using fluorescently labeled DGEA peptide for visualization. In comparison to hMSCs, interaction of ND-DGEA with PC3 was much greater. Green represents fluorescence due to DGEA peptide. All images are shown with 40× magnification.

The ability of DGEA to specifically interact with metastatic prostate cancer cells was observed using PC3 and hMSC cells – which should have a lower expression of α_2_β_1_ integrins in comparison to PC3. Since PC3 is a bone metastatic cancer, hMSCs were selected for use as a model for normal bone cells. Cell lines were incubated with 10 µg/mL ND-DGEA for 32 h, washed three times with PBS to remove any unattached or internalized conjugates, and imaged with fluorescent microscopy since the DGEA peptide contained a FITC fluorescent label. The merged bright field and fluorescence images of hMSCs and PC3 cells show the representative interaction that was observed between the ND-DGEA conjugates and the PC3 cells or the hMSCs (Figure 5). In the randomly selected image fields (*n* = 6), it was observed that PC3 cells had 4 times more ND-DGEA conjugates attached or uptaken in comparison to the hMSCs. This also indicated that the expression of α_2_β_1_ integrins was higher in PC3 cells, as suggested by several researchers [31–33].

### Efficacy of the ND-DGEA+DOX system

**Figure 6:**
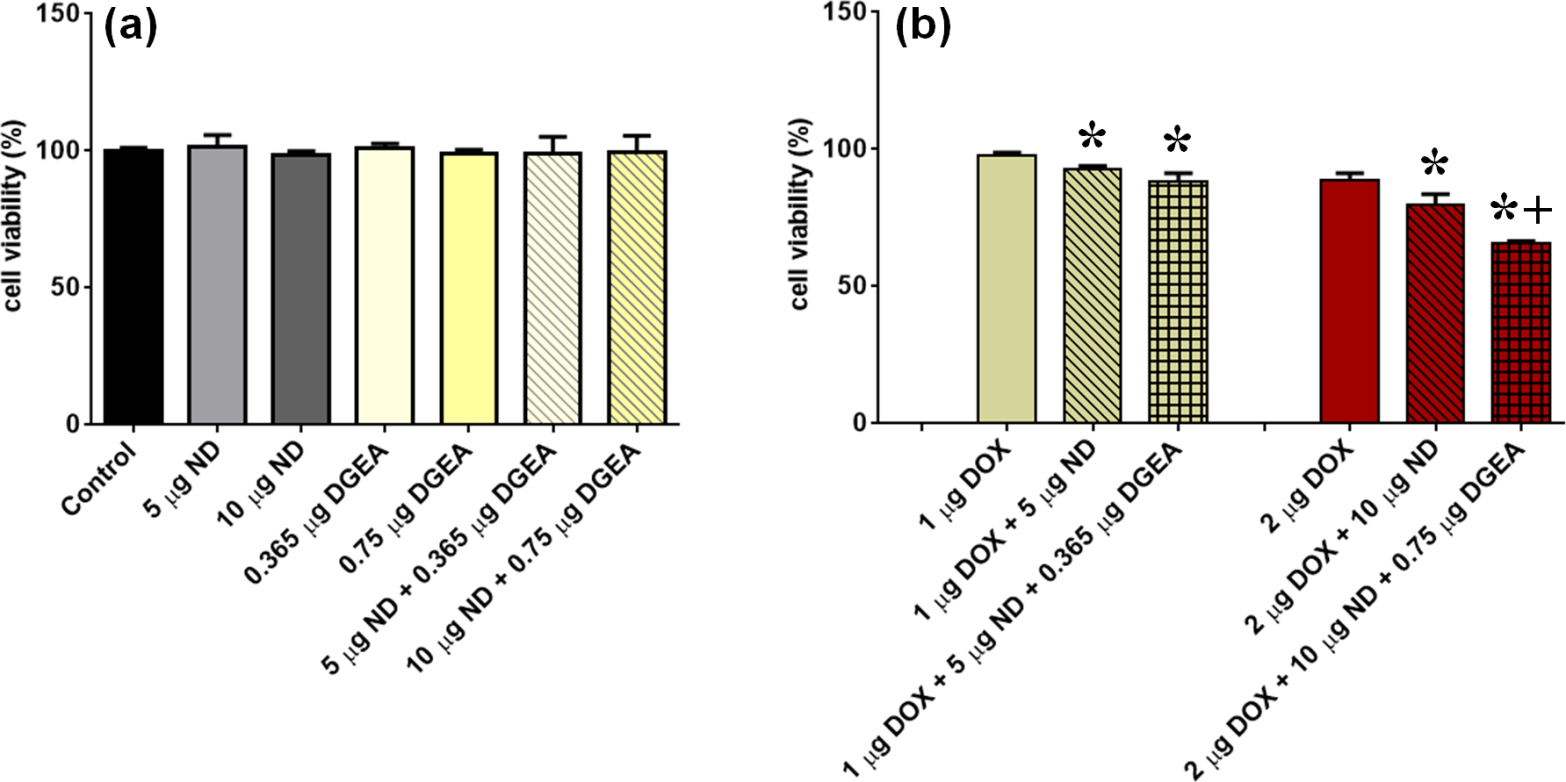
MTS assay of cell viability after 32 h exposure to various treatments. PC3 cells were treated with concentrations per mL of (a) ND, ND-DGEA, and DGEA peptide and (b) DOX, ND-DOX, and ND-DGEA-DOX. Symbols indicate significant difference (p < 0.05) when compared to drug alone (*) and ND-DOX system (**+**) at same dose.

After confirmation that DGEA peptide facilitates increased interaction with PC3, the effects of this α_2_β_1_ targeting system were investigated for DOX drug delivery enhancement. To ensure that the NDs, DGEA, and ND-DGEA did not induce toxic effects, PC3 cells were first exposed to these treatments for 32 h, and MTS cell viability assay was performed. As shown in Figure 6a, there were no significant differences in cell viability for any of the treatments; the cell viabilities for NDs, DGEA, and ND-DGEA conjugates were all comparable to the control.

With the demonstration that the individual drug delivery components did not elicit toxicity, PC3 cells then were exposed to no treatment (control) and various concentrations of free DOX, ND-DOX, and ND-DGEA-DOX for 32 h. Figure 6b summarizes the results of the MTS cell viability assay. The ND-DGEA+DOX systems caused significantly higher cell death than comparable DOX doses alone; cell death increased from 2.5% to 12% and 11% to 34% for 1 µg/mL and 2 µg/mL DOX doses, respectively, when ND-DGEA conjugates were utilized. Although the ND-DOX systems displayed significantly better efficacy than free DOX, the ND-DGEA+DOX system with 2 µg/mL DOX had superior efficacy to its comparable ND-DOX system (20% cell death) and displayed the best results of all treatments. These results were consistent with previous reports on the ability of ND to improve the efficacy of DOX [26–28] and targeted NDs to enhance the efficacy of various chemotherapeutics [16,21,24].

Since the ND-DGEA+DOX system had superior efficacy and improved drug delivery, there may be a synergistic effect in using both the NDs and DGEA. Several researchers have confirmed that integrin targeting increases drug delivery and ultimately efficacy [38–40]. Liang et al. demonstrated that DOX-loaded micelles can efficiently use the tumor-targeting function of RGD sequence to deliver the drug into HeLa cells [38]. Tian et al. showed that iRGD exosomes delivered DOX specifically to tumor tissues and inhibited tumor growth without overt toxicity [39]. Zhou et al. proved that graphene oxide functionalized with an α_V_β_3_ integrin mono-antibody selectively transports DOX into the targeted cancer cells, where then DOX is released into the cytoplasm and moved into the nucleus leading to a high therapeutic efficiency [40].

Even though the ND-DGEA+DOX system had superior efficacy and improved drug delivery, it is assumed that DOX has to detach in order to maintain the functionality of its mechanism of action. The mechanism of action for DOX involves the crosslinking of DNA, inhibiting DNA replication. In release studies (not shown), 85% of DOX was retained to the ND surface. However, enhanced efficacy was observed without DOX detachment. It is plausible that DGEA increased the interaction between the NDs and PC3 cells, and ND-mediated systems were endocytosed into cells increasing intracellular drug concentrations. As a result of the increased intracellular drug concentrations, DOX efficacy was enhanced. The quantitative analysis of DOX release kinetics and cellular internalization are limitations of this study and should be the subject of future studies.

## Conclusion

In this study, a novel targeted drug delivery system consisting of NDs (drug delivery vehicle), DGEA peptide (targeting agent), and DOX (cancer drug) was developed. This targeted drug delivery system is an important advance in the field of nanotechnology due to its implications for the fields of cancer therapeutics and drug delivery, especially for bone metastatic prostate cancer. Successful preparation of the ND-DGEA conjugates and ND-DGEA+DOX system was confirmed with transmission electron microscopy, hydrodynamic size measurements, and zeta potential measurements. The interaction of ND-DGEA conjugates with α_2_β_1_ integrins was confirmed using hMSCs (control) and PC3 cell lines. Here for the first time, it was demonstrated that α_2_β_1_ targeting with DGEA peptide and NDs improves the efficacy of DOX as the ND-DGEA+DOX systems improved the efficacy of 1 µg/mL DOX and 2 µg/mL DOX to achieve 12% and 34% cell death, respectively. Although there was an increase in ND-DGEA interaction with the PC3 cells in comparison to hMSCs that confirmed targeting, the exact cellular mechanisms for the superior efficacy of the ND-DGEA+DOX system have not been confirmed. Yet, by demonstrating that DGEA targeting enhances therapeutic efficacy, research progresses towards the realization of clinical therapies that selectively target cancers decreasing toxicity and drug doses, while improving treatment efficacies.
